# [6]-Paradol suppresses proliferation and metastases of pancreatic cancer by decreasing EGFR and inactivating PI3K/AKT signaling

**DOI:** 10.1186/s12935-021-02118-0

**Published:** 2021-08-10

**Authors:** Xueyi Jiang, Jie Wang, Peng Chen, Zhiwei He, Jian Xu, Yankun Chen, Xinyuan Liu, Jianxin Jiang

**Affiliations:** 1 Department of Hepatobiliary Surgery, Renmin Hospital of Wuhan University, 99 Ziyang Road, Wuhan City, Hubei Province 430060 People’s Republic of China; 2 Department of Hepatic-Biliary-Pancreatic Surgery, The Affiliated Hospital of Guizhou Medical University, Guiyang, Guizhou China

**Keywords:** [6]-Paradol, Pancreatic cancer, Proliferation, Metastasis, EGFR

## Abstract

**Background:**

The underlying mechanism behind the tumorigenesis and progression of pancreatic cancer is not clear, and treatment failure is generally caused by early metastasis, recurrence, drug resistance and vascular invasion. Exploring novel therapeutic regimens is necessary to overcome drug resistance and improve patients outcomes.

**Methods:**

Functional assays were performed to investigate the role of [6]-Paradol (6-P) in proliferation and metastasis of pancreatic cancer in vitro and in vivo. The interaction between EGFR and 6-P was tested by KEGG enrichment analysis and molecular docking analysis. qRT-PCR was performed to detect the mRNA expression of EGFR in 6-P treated groups. Involvement of the PI3K/AKT pathway was measured by western blotting.

**Results:**

6-P significantly suppressed pancreatic cancer cell proliferation and metastasis. KEGG enrichment analysis and molecular docking analysis suggested that there existed certain interaction between EGFR and 6-P. In addition, 6-P obviously decreased EGFR protein expression level but did not change the mRNA expression level of EGFR. 6-P could induce degradation of EGFR through decreasing the protein stability of EGFR and enhancing the ubiquitin-mediated proteasome-dependent degradation, 6-P-mediated EGFR degradation led to inactivation of PI3K/AKT signaling pathway. However, ectopic expression of EGFR protein resulted in resistance to 6-P-mediated inactivity of PI3K/AKT signaling and inhibition of malignant phenotype of pancreatic cancer. Inversely, erlotinib could enhance the 6-P-mediated anticancer activity.

**Conclusion:**

Our data indicated that 6-P/EGFR/PI3K/AKT signaling axis might become one of the potential therapies for the treatment of pancreatic cancer.

**Supplementary Information:**

The online version contains supplementary material available at 10.1186/s12935-021-02118-0.

## Introduction

Pancreatic cancer is the third highest in cancer-related deaths in the US and has the lowest 5-year survival rates (less than 10%), carrying an extremely poor survival conditions in digestive malignant cancer [1]. Although the laparoscopic pancreaticoduodenectomy is widely used in pancreatic surgery for reducing the recovery time and the modern chemotherapeutic regimens significantly improve the prognosis, the recurrence rate of pancreatic cancer is still high and the available treatment options are limited, resulting in unsatisfied clinical outcomes [2]. Surgical resection offers opportunities for curing the pancreatic cancer, however, most of patients lose their surgical chances due to insidious onset and rapid progression in early stage [3]. Chemotherapy and radiotherapy are main supplemental therapeutic strategies in advanced stage of pancreatic cancer. The first-line chemotherapy gemcitabine combining with other chemotherapeutic agents, including albumin-bound paclitaxel or cisplatin, are often used to treat patients with pancreatic cancer. However, patients commonly develop drug resistance and metastasis in the later stage, resulting in treatment failure. Therefore, it is particularly necessary to develop new therapeutic regimens, including chemotherapy, radiotherapy, bio-targeted drugs and traditional Chinese medicine.

In recent years, great progress has been made in the anti-tumor research of natural compounds and their derivatives. Ke et al. reported that the extracts of foeniculum vulgare seed could promote lung cancer cells apoptosis by suppressing the Bcl-2 expression [4]. ZM-32, one of muscone derivative, had been proved to exert an inhibitory function in breast tumor angiogenesis through effectively blocking the interaction between HuR and VEGF [5]. A typical flavonoid compound, baicalein, extracted from the root of Scutellaria baicalensis, inhibited lung cancer cell proliferation via inducing degradation of MAP4K3. The underlying molecular mechanism was that baicalensis could directly interact with MAP4K3 to decrease its protein stability and promote its ubiquitination modification [6].

Ginger (Zingiber officinale) is one of the most natural dietary ingredient, containing several pungent constituents, including ginerols, paradols, shogaols and gingerdiols [7]. In addition to being widely used as a flavoring ingredient, ginger roots are also applied in traditional Eastern herbal remedies for symptoms such as the common cold, digestive ailments, rheumatism, neuralgia, colic and motion sickness [8]. Recent researches indicate that the extracts of ginger exert multiple biologic functions such as anti-melanogenesis, anticancer, antioxidant and anti-inflammatory properties [9–11]. Previous study suggested that [6]-Shogaol (6-S) suppressed lung cancer cells proliferation through inhibiting the activity of AKT kinase and inducing cell cycle arresting at G1 or G2/M phase [12]. Another extract [6]-Gingerol (6-G) was reported to possess anti-proliferative and angiogenesis in colorectal cancer by decreasing the concnetration of VEGF [13]. Mariadoss et al. demonstrated that [6]-Paradol (6-P) effectively prevented mouse skin carcinogenesis process [14]. Additionally, the viability and proliferation of human promyelocytic leukemia could be inhibited by 6-P mediated cytotoxic activity [15].

In this study, we identified an ingredient of ginger, 6-P, as a potential candidate for the anti-tumor compound and therapy of pancreatic cancer. We discovered that 6-P exerted anti-pancreatic cancer activity by decreasing the expression of EGFR and inhibiting the activity of AKT signaling. Our data indicated that 6-P might serve as one of the potential therapies for the treatment of pancreatic cancer.

## Materials and methods

### Experimental drugs, reagents and antibodies

P (No. HY-14617), MG-132 (No.HY-13259) and erlotinib (No. HY-50896) were purchased from the MedChemExpress (MCE, USA). Cycloheximide (CHX, No. 66-81-9) were purchased from the Merck (USA). The Primer sequences, including EGFR, GAPDH were purchased from Ribobio (China). Flag-labeled EGFR overexpressed plasmid and HA-labeled ubiquitin plasmid were constructed and extracted from Genechem (China). Dulbecco's modified eagle medium (DMEM) and fetal bovine serum (FBS) were purchased from Gibco (USA). Cell counting kit-8 (CCK-8) was purchased from Dojindo (Japan). Matrigel was purchased from BD Biosciences (USA). PCR Reagents used include TRIzol (Invitrogen, USA), HiScript® III 1st Strand cDNA Synthesis Kit (+ gDNA wiper) (Vazyme, China), ChamQ Universal SYBR qPCR Master Mix (Vazyme), Lipofectamine 3000 (ThermoFisher, USA). Western blot Reagents used include BCA protein assay kit (Boster, China); enhanced chemiluminescent Kit (ABclonal Technology, China) Antibodies used include EGFR, AKT, PI3K, GAPDH rabbit antibody (Proteintech, China); p-AKT, p-PI3K rabbit antibody (CST, USA). HRP-labeled goat anti-rabbit IgG (Boster, China).

### Cell culture and transfection

Human pancreatic cancer cell lines MIA PaCa-2 and SW1990 were purchased from American Type Culture Collection. The two pancreatic cancer cells were cultured in DMEM with supplementary 10% FBS. The plasmids were respectively transferred into adherd pancreatic cancer cells with Lipofectamine 3000 Transfection Reagent. After 6 h of incubation, wash with PBS and change the medium. The transfected effectiveness was evaluated by PCR analysis.

### CCK-8

Cell viability was measured using CCK-8, and approximate 2 × 10^2^ MIA PaCa-2 and SW1990 cells were respectively seeded into 96-well plates. Different concentrations of 6-P were prepared in advance, including 0 μM, 20 μM, 40 μM, 80 μM, and respectively added into 96-well plates. After regular time points (0 h, 24 h, 48 h, 72 h) of incubation, 10 μl CCK-8 solution was respectively added into 96-well plates for another 3 h. Then, the absorbance at 450 nm was identified to evaluate the relative cell proliferation by a microplate reader.

### Plate colony formation

The ability of pancreatic cancer cells on colony formation was measured using plate colony formation assay. Approximate 1 × 10^3^ MIA PaCa-2 and SW1990 cells were respectively seeded into 6-well plates. Different concentrations of 6-P were prepared in advance, including 0 μM, 20 μM, 40 μM, 80 μM, and respectively added into 6-well plates for 7 days co-incubation. Then the 6-well plates needed be washed with PBS, fixed with 4% paraformaldehyde, stained with 1% crystal violet. Lastly, the stained 6-well plates were imaged and recorded with an HD Camera.

### Wound healing assay

The migrated ability of pancreatic cancer cells was measured by wound healing assay. Approximate 5 × 10^5^ MIA PaCa-2 and SW1990 cells were respectively seeded into 6-well plates until cells covered with the whole plates. Using 200 μl pipette tip drew a vertical line in 6-well plate and the wound was washed with PBS. The different concentrations of 6-P were respectively added into 6-well plate to co-incubate for 48 h. The wound healing images were captured using microscope.

### Transwell assay

The migrated and invasive ability of pancreatic cancer cells were measured by transwell assay. Approximate 200 μl suspension containing 1 × 10^4^ MIA PaCa-2 or SW1990 cells were respectively seeded into 8 μm filter pore size transwell inserts, the upper chambers which were covered with Matrigel. Approximate 600 μl DMEM with 20% FBS was added into the lower chambers. After 24 h of incubation, the cells on upper chambers were washed and removed, the cells on lower chambers were fixed with 4% paraformaldehyde and stained with 1% crystal violet. The stained lower chambers were imaged and recorded under a microscope using five randomized fields.

### PCR analysis

The total RNA was extracted from pancreatic cancer cells with Trizol Reagent, the RNA was reverse transcribed into cDNA using HiScript® III 1st Strand cDNA Synthesis Kit. Then the target genes were quantified according to their specific primer sequences via Bio-Rad RT-PCR System using ChamQ Universal SYBR qPCR Master Mix. GAPDH acted as an internal reference. The primer sequences as bellow: GAPDH, Forward: GGAGCGAGATCCCTCCAAAAT; Reverse: GGCTGTTGTCATACTTCTCATGG. EGFR, Forward: CCCACTCATGCTCTACAACCC; Reverse: TCGCACTTCTTACACTTGCGG.

### Western blot

Total proteins were extracted from cells using RIPA Lysis buffer and the concentration was measured using BCA method. The equal amount of protein (40 μg) was loaded and separated by SDS–polyacrylamide gel electrophoresis. After electrophoresis, the protein were transferred to PVDF membrane. Then, the membrane was blocked using defatted milk for 2 h. Subsequently, the specific primary antibody was added into a box to incubate with the membrane at 4℃ overnight. Then the membrane was washed with TBST and incubated with the secondary antibody at room temperature for 2 h. After washing with TBST, the protein band were evaluated and visualized using ECL reagents via Bio-Rad System.

### Xenograft tumor-formation assay and treatment

Ten female BALB/c mude mice (13–15 g) with 6 weeks of age were purchased from Huafukang Biotechnology Co., Ltd (China). SW1990 cells were prepared into cell suspension with germfree PBS. Approximately 200 μL PBS suspension containing 5 × 10^6^ cells was injected subcutaneously into the armpit of mice. 2 weeks after inoculation, all the mice were successfully tumor-bearing and were randomly divided into control group (n = 5) and 6-P treatment group (n = 5). The control group was given 100 μL of placebo ((10% dimethylsulfoxide (DMSO) + 90% corn oil)), however, the 6-P group was orally administered 6-P (dissolved in 10% DMSO + 90% corn oil) with 10 mg/kg into mice every day. The tumor volumes were measured once a week and calculated as V = (length × width^2^) × 0.5. After feeding for 5 weeks, the nude mice were euthanized by intravenous injection of pentobarbital (100 mg/kg). Subsequently, we removed the subcutaneous tumors that the wight of stripped tumors were measured using an electronic balance, then the tumors were embed it in wax block for immunohistochemical analysis.

### Statistical analysis

All data were expressed as mean ± standard deviation. SPSS 21.0 statistical software and Graphpad prism 8.0 were used to analyze data. Statistical significance was analyzed using the Student’s t test, one-way analysis of variance (ANOVA). Statistical significance was considered at a P value less than 0.05.

## Results

### Inhibition of 6-P on proliferation of pancreatic cancer cells

**Fig. 1:**
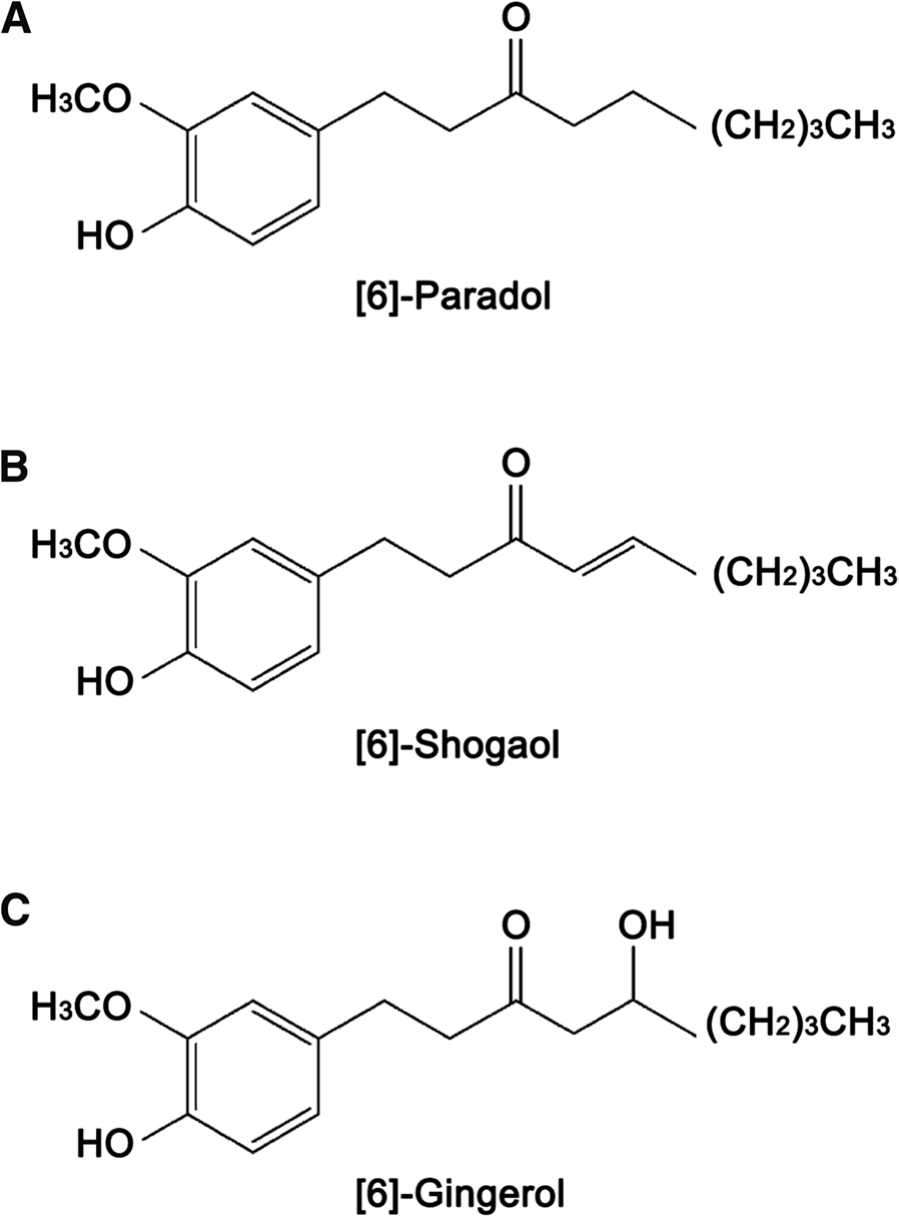
The chemical structural formulas of extracts of ginger. **A** [6]-Paradol. **B** [6]-Shogaol. **C** [6]-Gingerol

**Fig. 2:**
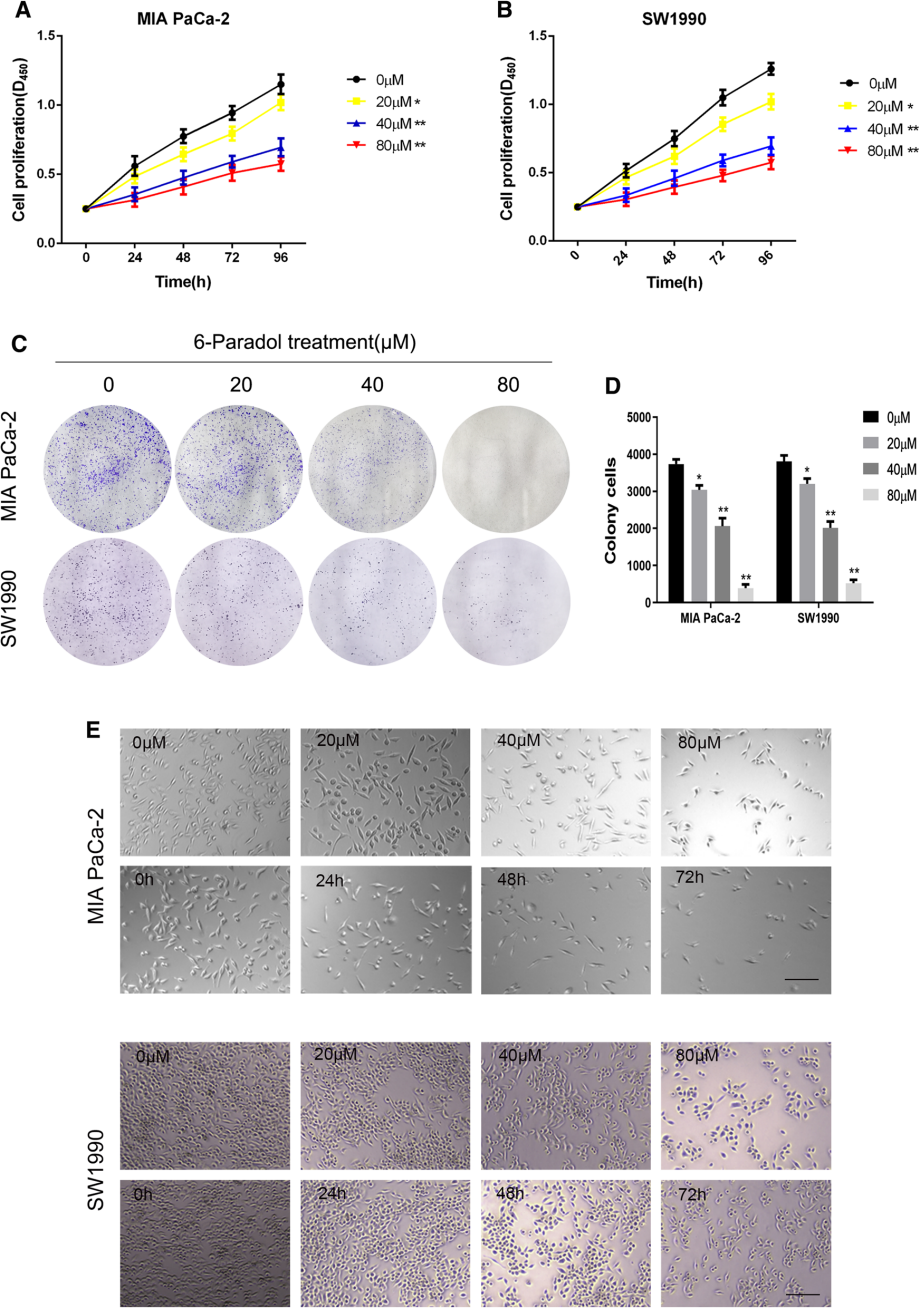
Inhibition of 6-Paradol on proliferation of pancreatic cancer cells. **A**, **B** CCK-8 assays were performed to measure proliferation ability of MIA PaCa-2 (**A**) and SW1990 (**B**), which were treated with different concentrations of 6-Paradol (0, 20, 40, 80 μM). **C**, **D** Colony formation assay was performed to test the cell colony ability in MIA PaCa-2 (**C**) and SW1990 (**D**), which were treated with different concentrations of 6-Paradol (0, 20, 40, 80 μM). **E**, **F** The pancreatic cancer cells were treated with different concentrations of 6-P for 48 h (0, 20, 40, 80 μM) or the same concentration of 6-P for different time frames (0, 24, 48, 72 h). And the morphological changes of MIA PaCa-2 **E** and SW1990 **F** were captured using a phase contrast microscope (scale bar: 200 μm). *p < 0.05, **p < 0.01

The chemical structure of 6-P and its derivatives 6-G and 6-S was displayed in Fig. 1. To investigate the effect of 6-P on proliferation of pancreatic cancer, pancreatic cancer cell lines MIA PaCa-2 and SW1990 were treated with different concentrations of 6-P for 48 h (0, 20, 40, 80 μM) or the same concentration of 6-P for different time frames (0, 24, 48, 72 h). First, CCK-8 assay was performed to evaluate the effect of 6-P on pancreatic cancer cell viability. The results suggested that cell viability significantly decreased with increasing 6-P concentration in MIA PaCa-2 and SW1990 (Fig. 2A, B). In addition, cell colony formation assay indicated the same results that the number of cell colonies was obviously inhibited by culturing with different concentrations of 6-P and the 80 μM concentration showed a highest inhibited effect on cell colonies (Fig. 2C, D). To further evaluate the cytotoxicity and anti-proliferation of 6-P, we used a phase contrast microscope to observe and capture the morphological changes of pancreatic cancer MIA PaCa-2 and SW1990 treated with 6-P. The results uncovered that 6-P caused adherent pancreatic cells to become round, shrink, wiredrawing and separate from the bottom of the culture plates, indicating a significant apoptosis state, especially in concentration of 80 μM or treating with 72 h (Fig. 2E, F).

### Inhibition of 6-P on migration and invasion of pancreatic cancer cells

**Fig. 3:**
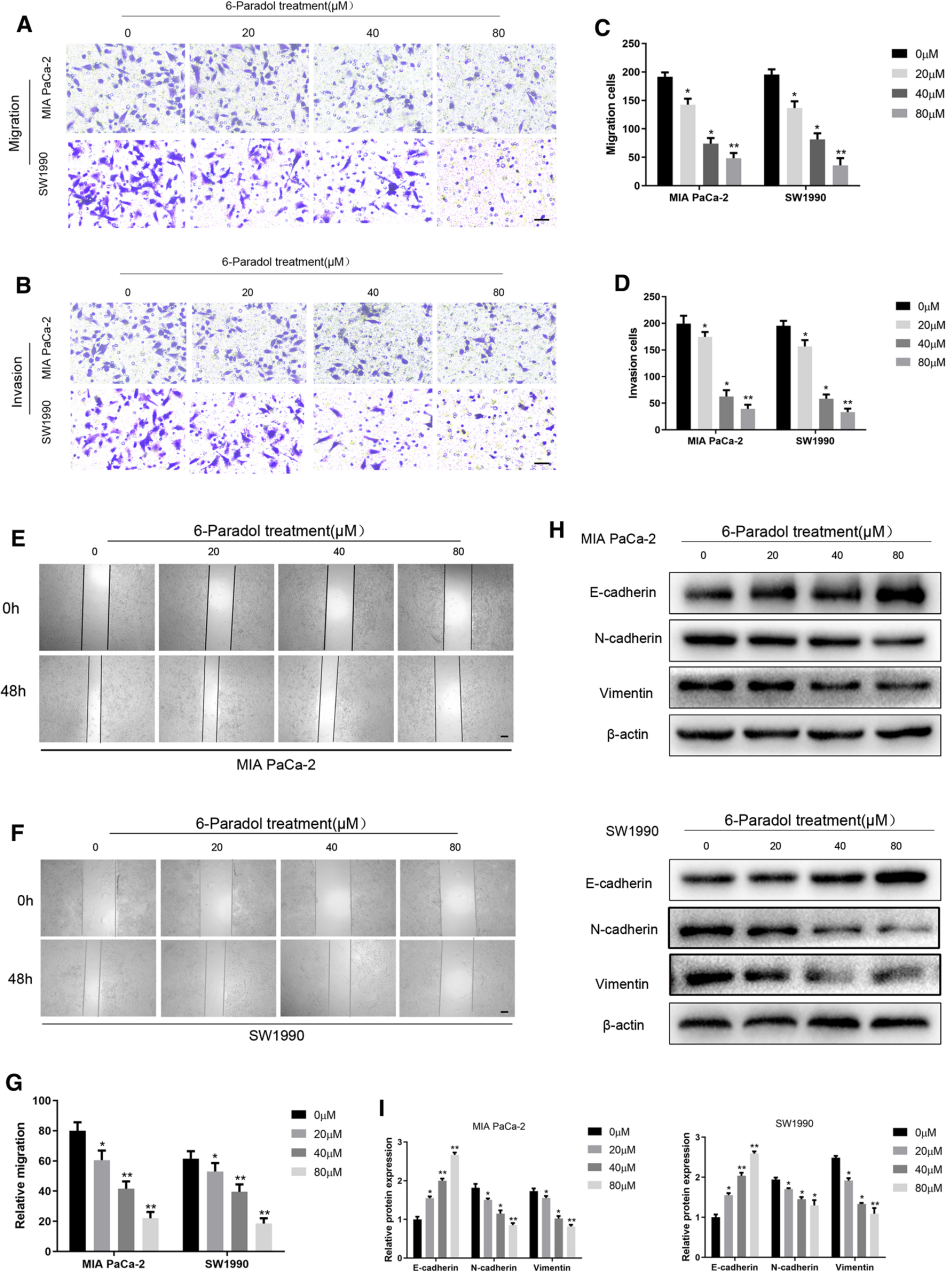
Inhibition of 6-Paradol on migration and invasion of pancreatic cancer cells.** A**–**D** Transwell assays were performed to evaluate the migration and invasion abilities of pancreatic cancer cells and the cells were treated with different concentrations of 6-Paradol (0, 20, 40, 80 μM). Migration abilities were evaluated without Matrigel (**A**) and invasion abilities were evaluated with Matrigel (**B**) (scale bar: 100 μm). The statistical chart of migration cells (**C**) and invasion cells (**D**) in different concentrations of 6-Paradol. **E**–**G** Wound healing assays were performed to further measure the migrated abilities in MIA PaCa-2 and SW1990 (scale bar: 400 μm). **H**, **I** Western blot assays were used to analyze the effect of 6-Paradol on EMT. *p < 0.05, **p < 0.01

To further validate whether 6-P had the inhibitory effect on migration and invasion of MIA PaCa-2 and SW1990, transwell assay and wound healing assay were performed to evaluate to migrate and invasive ability. The migration and invasion significantly decreased in the concentration of 40 and 80 μM compared with 0 μM, revealing that 6-P could also partly suppress the metastasis of pancreatic cancer cells (Fig. 3A–G). In addition, we tested the epithelial-mesenchymal transition (EMT) using western blot assay to detect the protein levels of E-cadherin, N-cadherin and Vimentin. The results demonstrated that the expression of E-cadherin gradually rose with the increasing concentration of 6-P. Conversely, the expression of N-cadherin and Vimentin gradually reduced with the increasing concentration of 6-P (Fig. 3H, I, Additional File 1). The results suggested an inhibited function of 6-P on EMT of pancreatic cancer cells.

### 6-P interacts with EGFR to exert suppression functions on proliferation and metastasis of pancreatic cancer cells

**Fig. 4:**
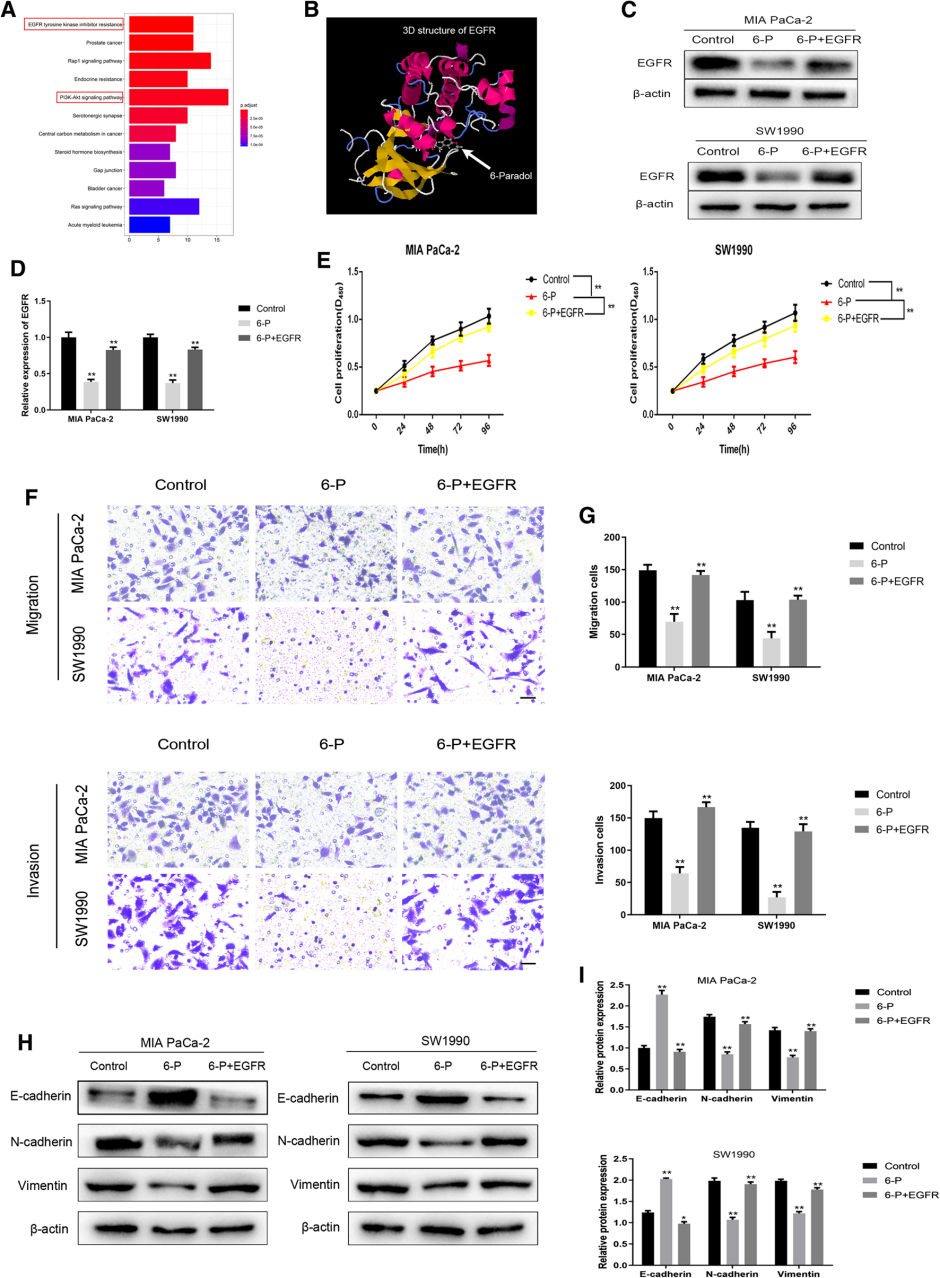
6-Paradol interacts with EGFR to exert suppression functions on proliferation and metastasis of pancreatic cancer cells. **A** PI3K-AKT signaling pathway and epidermal growth factor receptor (EGFR) tyrosine kinase inhibitor resistance were correlated with 6-Paradol using KEGG pathway enrichment analysis. **B** The 3D structure of molecular docking between 6-Paradol and EGFR. **C**, **D** Western blot assays were performed to measure the effect of 6-Paradol (80 μM) on EGFR protein levels. **E**–**G** Gain-Functional experiment was performed to measure the effect of 6-Paradol (80 μM) on proliferation and migration of 6-P + EGFR group in MIA PaCa-2 and SW1990 (scale bar: 100 μm). **H**, **I** Western blot was applied to investigate effect of 6-P combined with EGFR on EMT transition in MIA PaCa-2 and PANC-1. **p < 0.01

In order to explore the potential binding target of 6-P, bioinformatics methods were performed to predict the underlying protein site. First, we downloaded the 3-dimensional structure file of the compound 6-P from PubChem Compound Search database (https://pubchem.ncbi.nlm.nih.gov/). Then, we transeferred the data to SwissTargerPrediction software for predictive analysis and obtained the target protein of the compound 6-P. Subsequently, KEGG pathway enrichment analysis was performed to figure out the involved signaling pathway of these underlying target protein by DAVID database (https://david.ncifcrf.gov/). Finally, we set the standard for judging significant enrichment of pathways with a P value less than 0.05, and the top 12 signal pathways with enrichment number were visualized using R language with clusterProfilerKEGG package. The results of KEGG pathway enrichment analysis indicated that 6-P was significantly correlated with PI3K-AKT signaling pathway and epidermal growth factor receptor (EGFR) tyrosine kinase inhibitor resistance (Fig. 4A). Interestingly, molecular docking analysis with 6-P on the 3-dimensional structure of EGFR suggested that there was an interaction between 6-P and EGFR (Fig. 4B). Combined with KEGG results, we hypothesized that 6-P might occupy key sites of EGFR molecular structure to exert biological regulation functions. Subsequently, western blot assay was performed to detect EGFR expression of pancreatic cancer cells treated with 6-P. The results confirmed that 6-P could decrease expression of EGFR and the inhibition of 6-P on EGFR expression could be partly rescued with supplementary EGFR (Fig. 4C, D, Additional File 1). In addition, we further evaluated the proliferation and metastasis of pancreatic cancer cells treated with 6-P after adding EGFR plasmid to upregulate EGFR expression. The results suggested that the cell proliferation, migration and invasion could also be partly rescued with supplementary EGFR (Fig. 4E–G). And western blot results revealed upregulation of EGFR could reverse 6-P mediated-inhibition of EMT process (Fig. 4H, I, Additional File 1).

### 6-P-mediated ubiquitination degradation of EGFR leads to inactivate PI3K/AKT signaling pathway

**Fig. 5:**
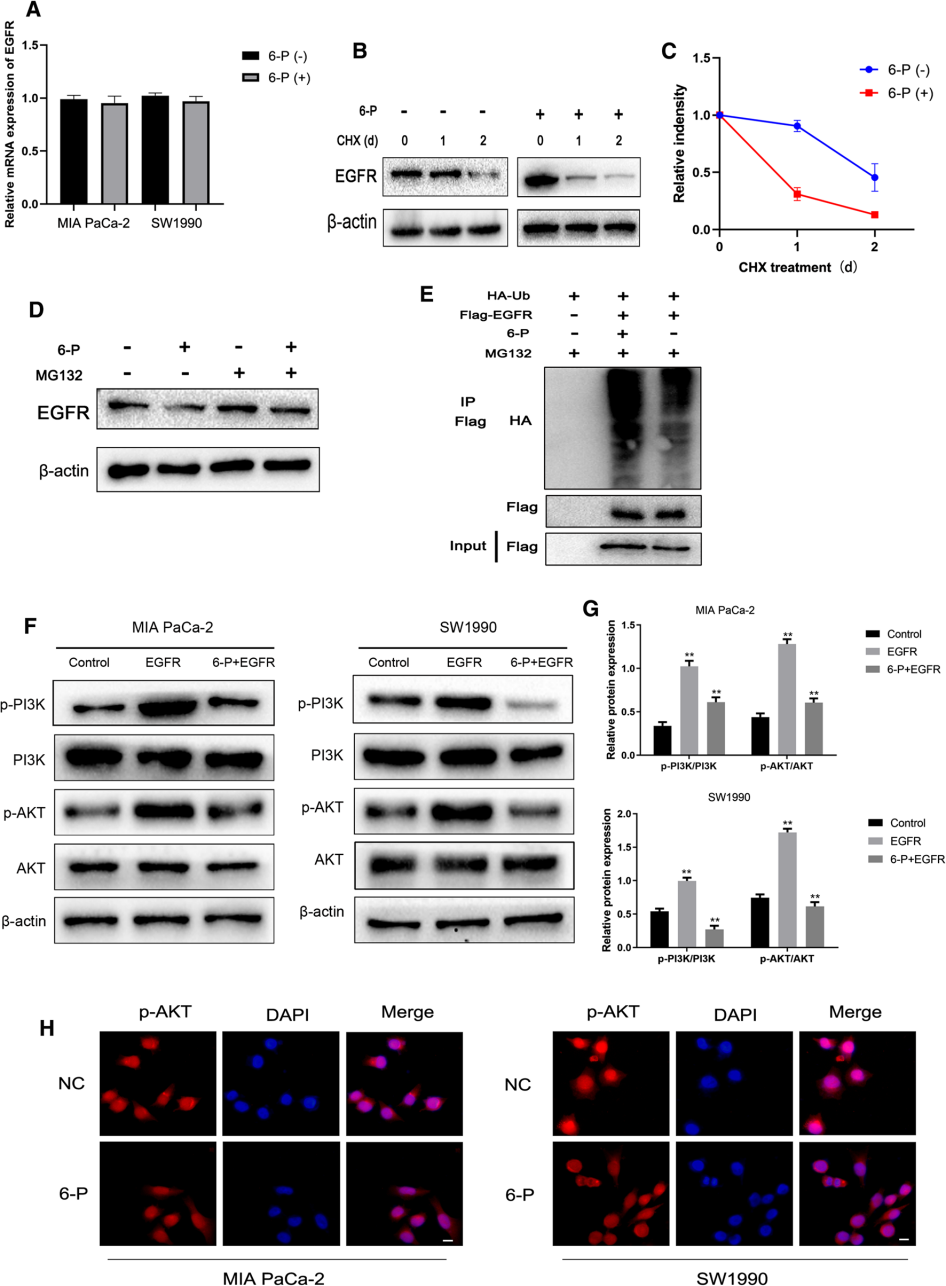
6-Paradol-mediated ubiquitination degradation of EGFR leads to inactivate PI3K/AKT signaling pathway. **A** The effect of 6-Paradol on EGFR mRNA by PCR analysis. **B**, **C** The effect of 6-Paradol on protein stability of EGFR was measured using 293 T cells treated with CHX for 1 h or 2 h. **D** A proteasome inhibitor, MG-132 (5 μM), was used to evaluate whether 6-Paradol was involved in EGFR degradation by a proteasome-dependent route. **E** Co-immunoprecipitation and SDS-gel electrophoresis were performed to evaluate the levels of EGFR ubiquitination in 293 T cells. **F**, **G** Western blot assay was performed to measure the protein expression levels of PI3K/AKT signaling in MIA PaCa-2 and SW1990 cells treated with 6-Paradol and EGFR overexpressed plasmid. **H** Immunofluorescence analysis of p-AKT levels in the 6-P and NC groups in MIA PaCa-2 and SW1990 cells. **p < 0.01

To further investigate the underlying molecular mechanism of 6-P on EGFR, we firstly evaluated the mRNA expression levels of EGFR in pancreatic cancer cells treated with 6-P. The results indicated that there was no significant difference in MIA PaCa-2 or SW1990 cells (Fig. 5A). However, our data suggested 6-P downregulated the protein expression of EGFR. To figure out the reason why 6-P changed the protein expression, not the mRNA expression, 293 T cells were treated with CHX and 6-P for 1 h, 2 h to evaluate the protein stability. The results demonstrated that the de novo synthesis of EGFR in 6-P treatment group reduced more rapidly compared to 6-P non-treatment group, suggesting 6-P decreased the protein stability of EGFR (Fig. 5B, C, Additional File 1). Subsequently, we suspected that downregulated EGFR protein expression was the result of 6-P-involved proteasome-dependent degradation mechanism. To validate the suspicion, a proteasome inhibitor, MG-132 (5 μM), was used to evaluate whether 6-P was involved in EGFR degradation by proteasome-dependent route. The results confirmed that MG132 inhibited EGFR degradation (Fig. 5D, Additional File 1). Then HA-labeled ubiquitin and Flag-labeled EGFR plasmids were co-transferred into the 293 T cells and 6-P was added to treat the cells. Co-immunoprecipitation and SDS-gel electrophoresis were performed to evaluate the levels of EGFR ubiquitination. Interestingly, the treatment of 6-P significantly enhanced EGFR ubiquitination, indicating 6-P promoted proteasome-dependent degradation of EGFR via ubiquitin modification pathway (Fig. 5E, Additional File 1). Subsequently, we detected the PI3K/AKT signaling pathway which was one of the downstream pathways of EGFR to further validate the results of KEGG pathway enrichment analysis. The results suggested that EGFR could activate PI3K/AKT while the activity of PI3K/AKT signaling could be reversed by treating with 6-P, indicating 6-P negatively activate PI3K/AKT signaling pathway (Fig. 5F, G, Additional File 1). Immunofluorescence staining was used to analysis the expression and localization of p-AKT. MIA PaCa-2 and SW1990 cells treated with 6-Paradol showed obvious decrease of p-AKT in comparison with the NC groups (Fig. 5H).

### EGFR inhibitor enhanced 6-P mediated-inhibition effect on PI3K/AKT signaling activity

**Fig. 6:**
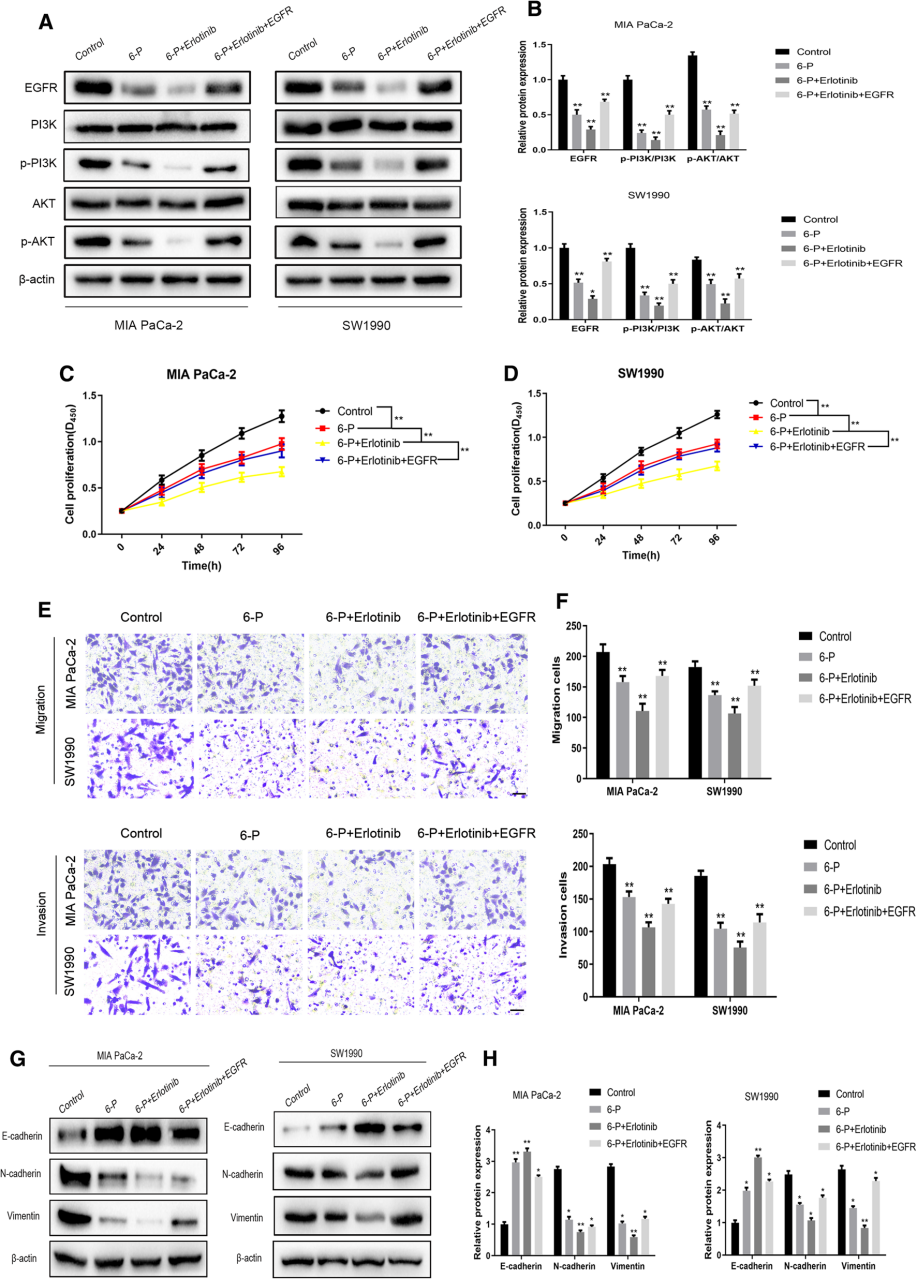
EGFR inhibitor enhanced 6-Paradol mediated-inhibition effect on PI3K/AKT signaling activity. **A**, **B** Western blot assay was performed to measure the protein expression levels of PI3K/AKT signaling in MIA PaCa-2 and SW1990 cells treated with 6-Paradol, EGFR overexpressed plasmid and Erlotinib (2 nM). **C**–**F** Gain- or Lose-Functional experiments were performed to measure the proliferation and migration abilities of pancreatic cancer cells, which were respectively treated with 6-Paradol, EGFR overexpressed plasmid and (or) Erlotinib (2 nM). **G**, **H** The protein expression changes of E-cadherin, N-cadherin and Vimentin in Control, 6-P, 6-P + Erlotinib and 6-P + Erlotinib + EGFR groups, respectively. *p < 0.05, **p < 0.01

To further confirm that 6-P mediated-EGFR degradation was involved in inhibitory effect on PI3K/AKT signaling, we respectively used EGFR overexpression plasmid and (or) EGFR inhibitor Erlotinib (2 nM) to regulate EGFR expression. The results verified that Erlotinib promoted 6-P mediated degradation of EGFR and inactivity of PI3K/AKT signaling, however, upregulation of EGFR expression could rescue the activity of PI3K/AKT signaling and the expression of EGFR (Fig. 6A, B, Additional File 1). Subsequently, gain- or lose-functional experiments were performed to evaluate the interaction between 6-P and EGFR on proliferation and metastasis of pancreatic cancer cells. The results revealed Erlotinib and 6-P had synergistic effects to exert inhibition on proliferation and metastasis of pancreatic cancer cells, which could be rescued by upregulation of EGFR expression (Fig. 6C–F). Meanwhile, Erlotinib combined with 6-P significantly inhibited EMT process and overexpressed EGFR removed the inhibitory effect on EMT process (Fig. 6G, H, Additional File 1).

### 6-P significantly suppressed tumor growth in vivo

**Fig. 7:**
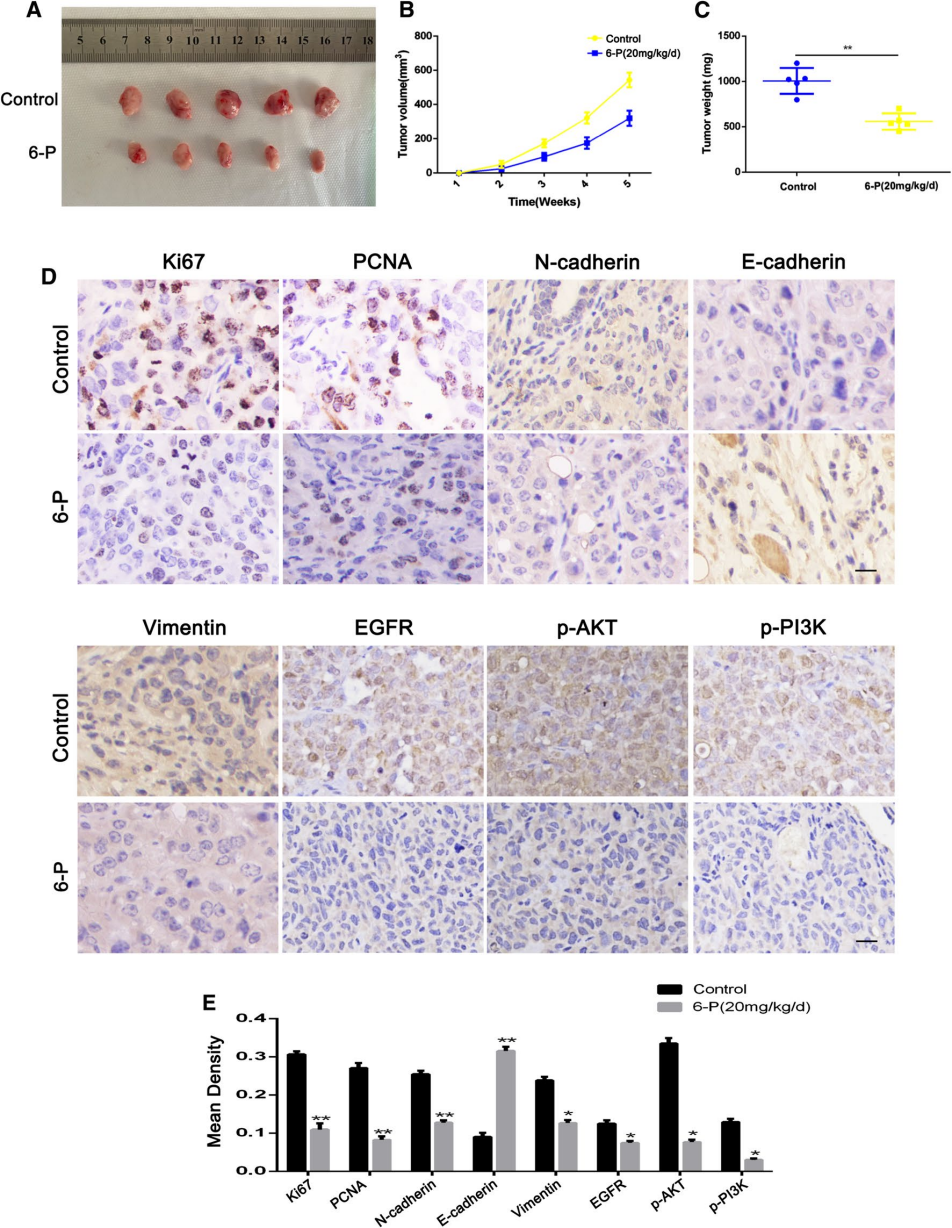
6-Paradol significantly suppressed tumor growth in vivo. **A** Tumor photographs of the subcutaneous xenografts in Control and 6-Paradol treatment groups, n = 5, cell line: SW1990. **B** Tumor volume of the subcutaneous xenografts in Control and 6-Paradol treatment groups. **C** Weight change curve in Control and 6-Paradol treatment groups. **D** IHC staining for Ki67, PCNA, EMT markers, EGFR, p-AKT and p-PI3K, and representative images of two pairs of subcutaneous xenograft tissue (100 ×). (Scale bar: 100 μm) *p < 0.05, **p < 0.01

To explore whether 6-P suppressed tumor growth in vivo, we constructed a subcutaneous tumorigenesis model in nude mice which were orally administered with 6-P (10 mg/kg/d). The results suggested the size of tumor was obviously smaller in 6-P treatment group compared with control groups, indicating a inhibitory function of 6-P on tumor growth (Fig. 7A). Interestingly, at 2 weeks of implantation, the tumor volume could be measured, thus we conducted a experiment for 6-P treatment on tumor-bearing mice. From 1 to 3 weeks after 6-P treatment, the tumor volumes was smaller in 6-P treatment groups and the stripped tumor weight was also lighter in 6-P treatment groups (Fig. 7B, C). Subsequently, IHC analysis was performed to detect relative expression of Ki67, PCNA, N-cadherin, E-cadherin, Vimentin, EGFR, phosphorylated-AKT and phosphorylated-PI3K. The results demonstrated E-cadherin expression was upregulated in 6-P treatment group and the rest of indexes were all downregulated, which were consistent with our previous results in vitro (Fig. 7D, E).

**Fig. 8:**
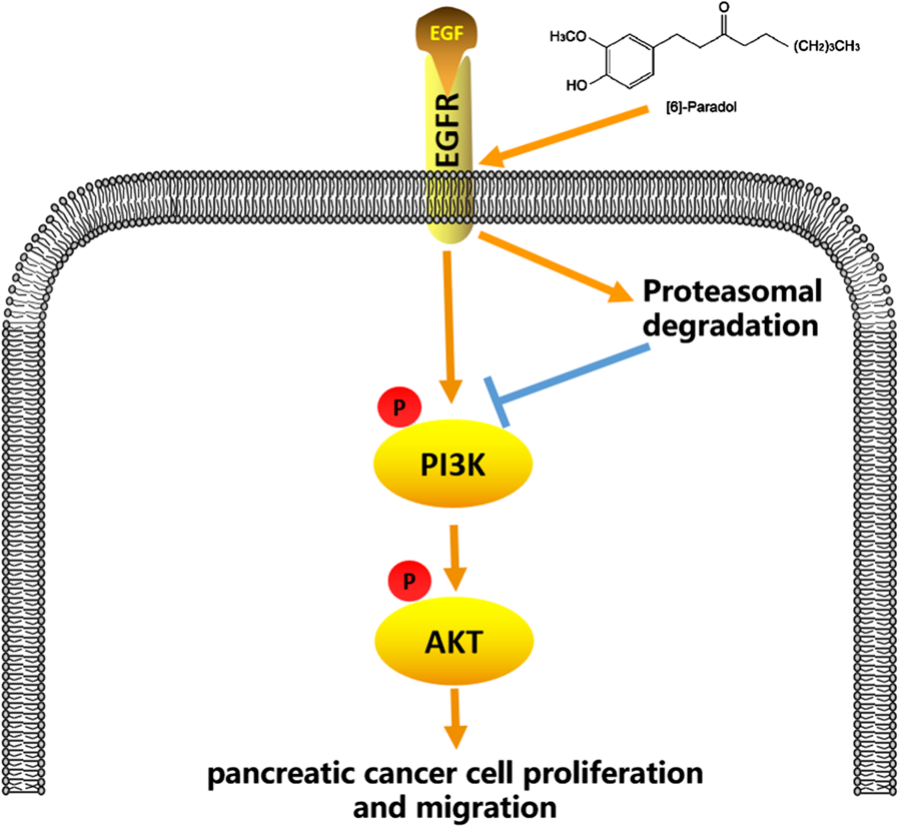
Schematic diagram of mechanism. [6]-Paradol inhibited pancreatic cancer cell proliferation and migration and inhibited the activation of PI3K/AKT signaling by targeting EGFR and enhancing EGFR degradation via a proteasome-dependent route

Overall, 6-P might functioned as an anti-tumor drug to inhibit pancreatic cancer cell proliferation and migration in vivo and in vitro by targeting EGFR, inducing EGFR degradation through decreasing the protein stability of EGFR and enhancing the ubiquitin-mediated proteasome-dependent degradation. Eventually, 6-P decreased EGFR expression and inhibited PI3K/AKT signaling to suppress tumor progress (Fig. 8).

## Discussion

The EGFR, a transmembrane protein receptor and an important member of tyrosine kinase receptors, is commonly elevated in cancers, engaging in multiple malignant functions such as aberrant activation of signaling, uncontrolled cell proliferation, vascular invasion and metastasis of the tumors [16]. Accumulating evidence indicates that EGFR expression is significantly correlated with pancreatic cancer, high expression of EGFR frequently suggests a poor prognosis [17]. Although many EGFR antibodies and inhibitors, including cetuximab, afatinib, osimertinib, erlotinib and gefitinib, have been applied to cancer treatment. The anticancer efficacy of them have negligible effects on patients with pancreatic cancer, especially in KRAS mutant pancreatic ductal adenocarcinoma [18]. Therefore, it’s urgent to carry out new drugs or novel combination therapy regimens to control pancreatic cancer process.

Recently, chemoprevention substances naturally existing in diets and medicinal plants have attracted widespread attentions [19]. 6-P, a phenolic compound in the rhizome of ginger, was reported that 6-P had potent anti-inflammatory activity, which exerted huge anticancer functions [20]. Previous study suggested 6-P and its derivative 6-G had the ability to reduce the viability of human promyelocytic leukemia HL-60 cell and induce tumor cell apoptosis, indicating 6-P possessed potential cytotoxic activity.[15] By decreasing STAT3 and inactivating NF-κB signaling, 6-P significantly reduced survival of prostate cancer cells [21]. In addition, 6-P could induce cell apoptosis in oral squamous carcinoma in a dose-dependent manner [8]. In our study, we first proposed that 6-P might be correlated with pancreatic cancer. Subsequently, we constructed tumor proliferation and metastasis model in vitro and in vivo, attempting to uncover the underlying molecular mechanism how 6-P affected pancreatic cancer procession. First, we evaluated the effect of 6-P on proliferation and metastasis of pancreatic cancer cells in different concentrations or in a same concentration for different application time. The results suggested 6-P inhibited pancreatic cancer cell proliferation and metastasis both in a time-dependent manner and a dose-dependent manner. Furthermore, tumor growth was obviously inhibited with 6-P treatment in vivo. Then, we discovered 6-P could reducing the protein expression of EGFR while did not change the mRNA expression of EGFR, suggesting 6-P had less effect at the transcriptional level of EGFR. Therefore, we further explored whether 6-P promoted EGFR degradation via proteasome-dependent degradation. The results suggested 6-P mediated post-translational modifications of EGFR via promoting EGFR ubiquitination, resulting in EGFR degradation. Additionally, we found 6-P reduced the activity of PI3K/AKT signaling via downregulation of EGFR, leading to decreasing abilities of cell proliferation and metastasis.

Ubiquitination plays an important role in protein localization, metabolism, function, regulation and degradation [22]. At the same time, it is also involved in the regulation of cell cycle, proliferation, apoptosis, differentiation, metastasis, gene expression, transcriptional regulation, signal transduction, injury repair, inflammation and immunity, and almost all life activities [23]. Zhang et al. found Ginsenoside compound K inhibited the proliferation of liver cancer via promoting the degradation of HIF-1α ubiquitination [24]. Liu et al. suggested that Honokiol had an anticancer function via directly interacting with keratin 18 protein in melanoma cells. The interaction between keratin 18 and Honokiol led to the degradation of keratin 18 by ubiquitination [25]. Another compound from ginger, 6-G, was also related with ubiquitination. The study indicated 6-G decreased the expression of USP14, which was an ubiquitin-specific peptidase mainly exerting inhibitory effect on ubiquitination. Decreased USP14 elevated the autophagosomes and reduced the survival of lung cancer cell [26]. In our data, 6-P mediated EGFR degradation through enhancing EGFR ubiquitination, resulting in inactivity of PI3K/AKT signaling.

Numerous investigations suggest hyperactivity PI3K/AKT signaling is associated with malignant phenotype of cancer and can accelerate cancer procession [27]. Totiger et al. found Urolithin A exerted anticancer effect in pancreatic cancer via downregulating phosphorylation of AKT [28]. Additionally, Amcp, one novel derivative of valepotriate significantly inhibited the PI3K/AKT signaling, suppressing the cell viability and Mcl-1 expression in pancreatic cancer cells [29]. In this study, we confirmed 6-P negatively regulated activity of PI3K/AKT signaling via decreasing EGFR.

## Conclusion

In conclusion, our data demonstrate that 6-P exerts anticancer effect though suppressing pancreatic cancer cell growth, viability, invasion and migration. Mechanistically, the inhibitory effect of 6-P mainly based on decreasing the expression of EGFR and inactivity of PI3K/AKT signaling via ubiquitination-mediated proteasomal degradation of EGFR. Therefore, 6-P might become a potential therapy for treatment of pancreatic cancer.

## Supplementary Information

**Additional file 1:** Orginal data.

## Data Availability

All data generated and analysed during this study are included in this published article are available on request.
