# Supplementary material for: [6]-Paradol suppresses proliferation and metastases of pancreatic cancer by decreasing EGFR and inactivating PI3K/AKT signaling
Source: Cancer Cell Int. 2021 Aug 10;21:420. doi: 10.1186/s12935-021-02118-0 (PMC8353760; doi:10.1186/s12935-021-02118-0)
Supplement: Supplementary file 1 — Additional file 1: Orginal data. [file 12935_2021_2118_MOESM1_ESM.pdf]

A

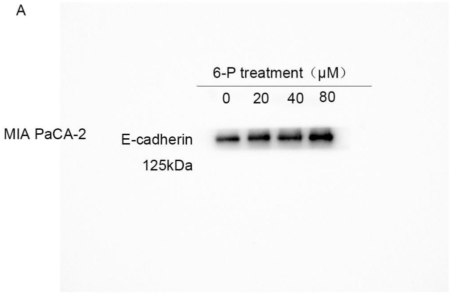

E

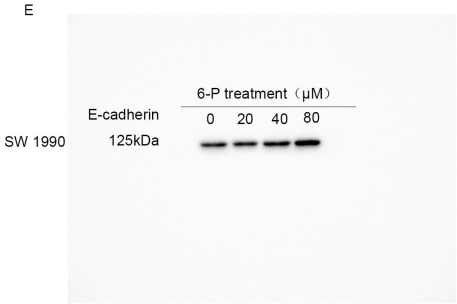

B

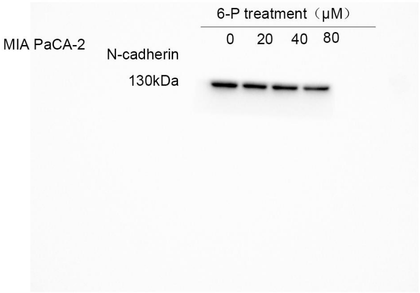

F

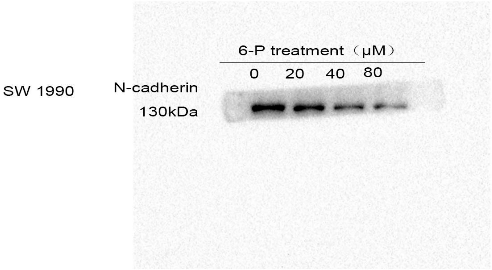

C

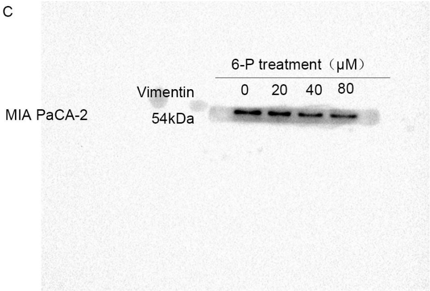

G

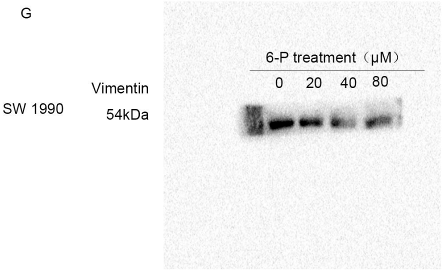

D

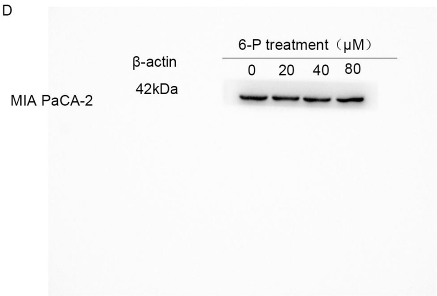

H

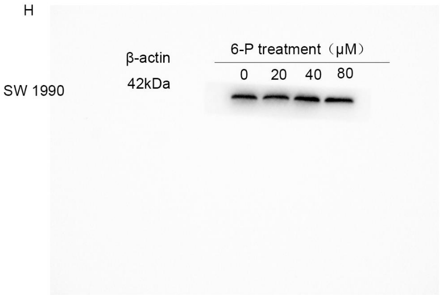

A-H represent western blot anailsis shown in Figure3 H

A

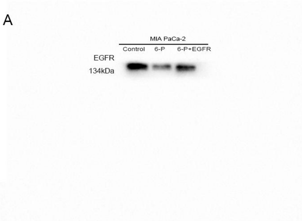

B

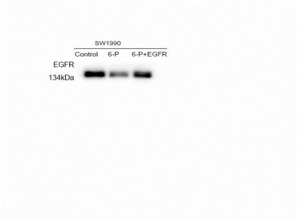

C

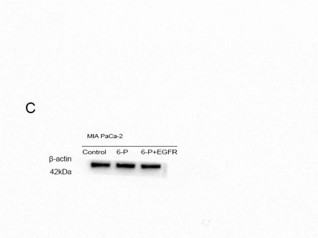

D

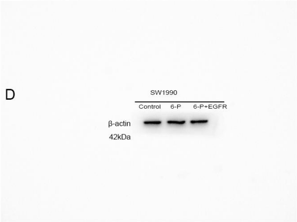

E

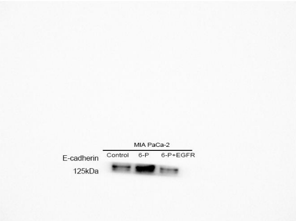

F

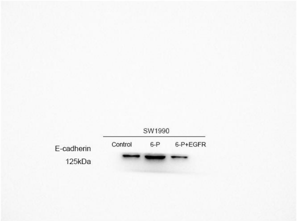

G

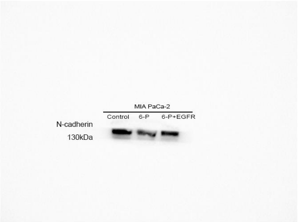

H

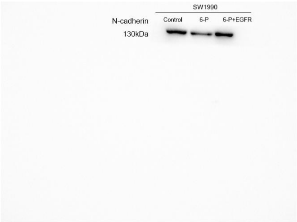

I

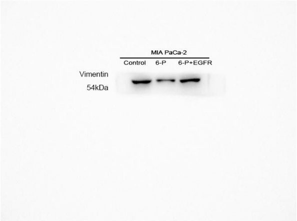

J

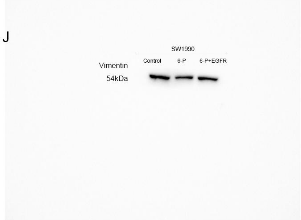

K

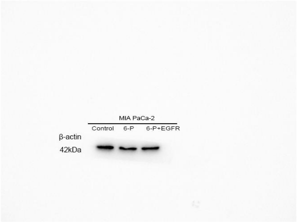

L

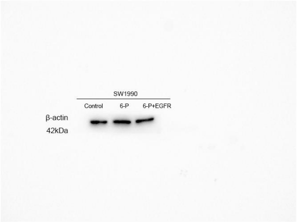

A,B,C,D represent western blot anailsis shown in Figure4 C

E-L represent western blot anailsis shown in Figure4 H

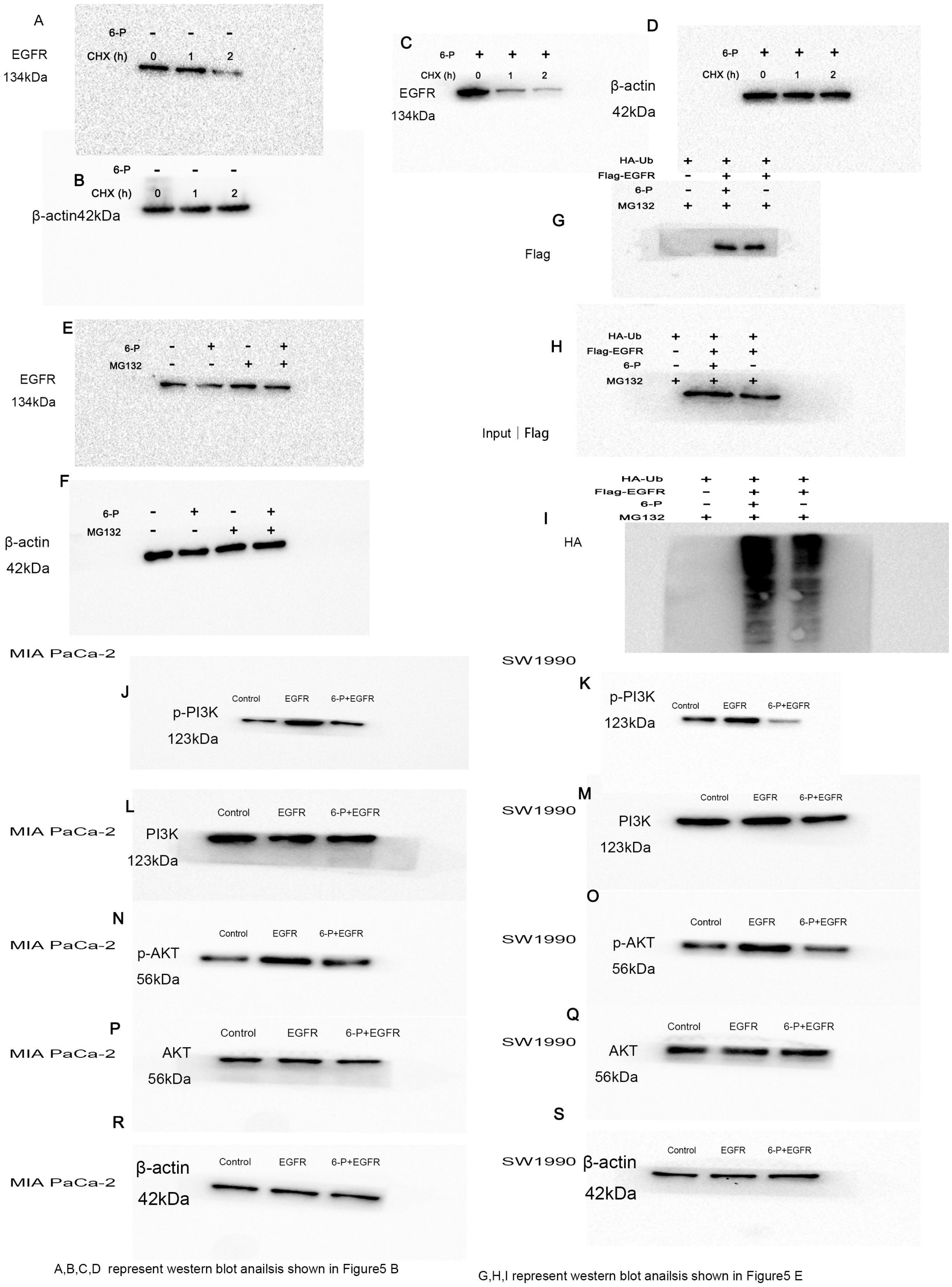

A,B,C,D represent western blot anailsis shown in Figure5 B

E,Frepresent western blot anailsis shown in Figure5 D

G,H,I represent western blot anailsis shown in Figure5 E

J-S represent western blot anailsis shown in Figure5 F

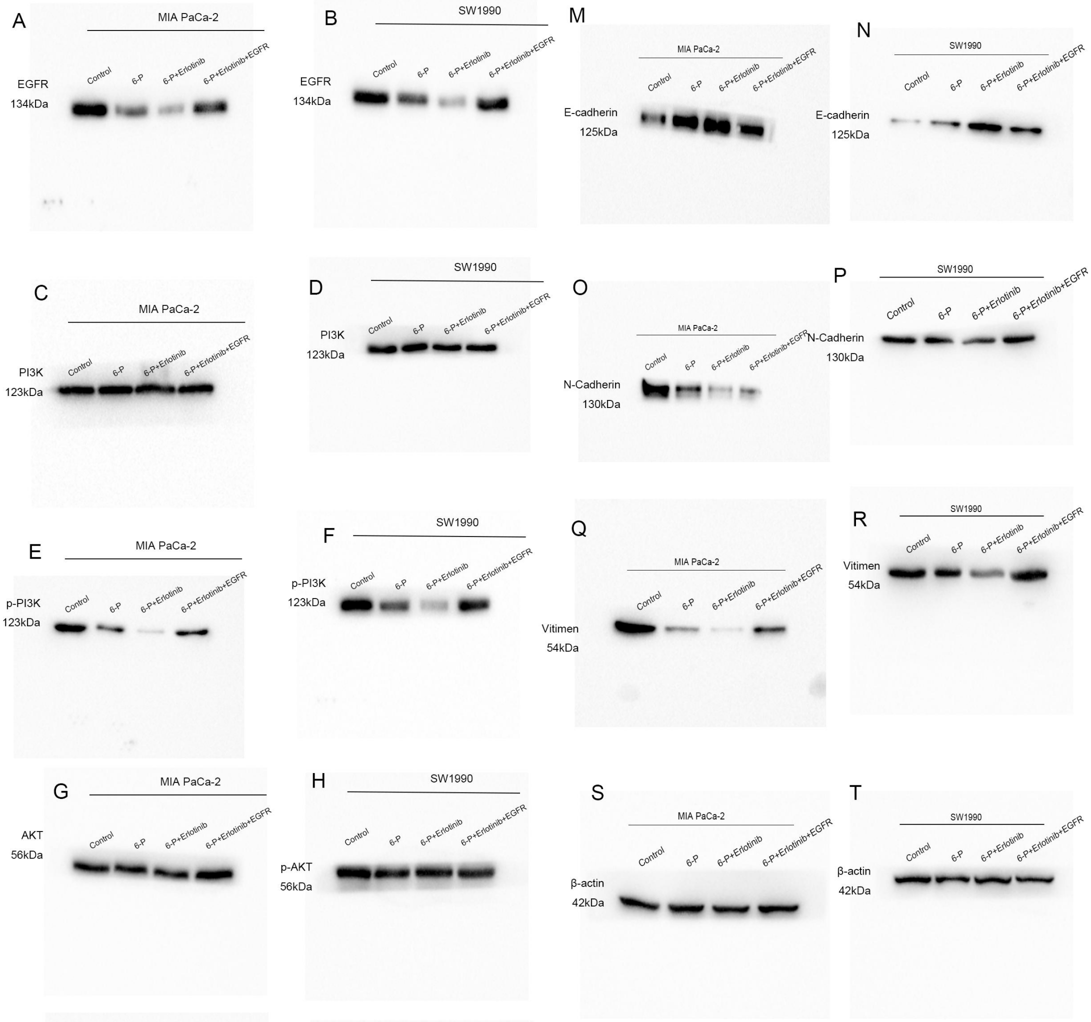

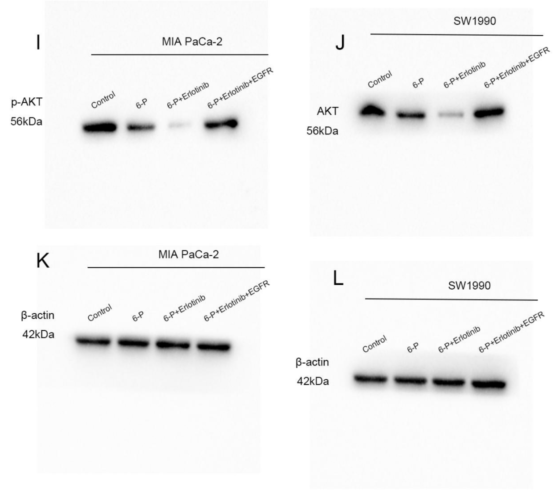

A-L represent western blot anailsis shown in Figure6 A

M-T represent western blot anailsis shown in Figure6 G
